# Artificial intelligence-based analysis of time-lapse images of sphere formation and process of plate adhesion and spread of pancreatic cancer cells

**DOI:** 10.3389/fcell.2023.1290753

**Published:** 2023-11-17

**Authors:** Yuuki Shichi, Fujiya Gomi, Yasuko Hasegawa, Keisuke Nonaka, Seiichi Shinji, Kimimasa Takahashi, Toshiyuki Ishiwata

**Affiliations:** ^1^ Division of Aging and Carcinogenesis, Research Team for Geriatric Pathology, Tokyo Metropolitan Institute for Geriatrics and Gerontology, Tokyo, Japan; ^2^ Department of Gastrointestinal and Hepato-Biliary-Pancreatic Surgery, Nippon Medical School, Tokyo, Japan; ^3^ Department of Veterinary Pathology, School of Veterinary Medicine, Nippon Veterinary and Life Science University, Tokyo, Japan

**Keywords:** pancreatic cancer, sphere, cell adhesion, migration, time-lapse analysis, epithelial-mesenchymal features, artificial intelligence, deep learning

## Abstract

**Background:** Most pancreatic cancers are pancreatic ductal adenocarcinomas (PDAC). Spherical morphology formed in three-dimensional (3D) cultures and the effects of anticancer drugs differ between epithelial and mesenchymal PDAC cell lines. In the human pancreas, cancer cells form 3D tumors, migrate to adjacent tissues, and metastasize to other organs. However, no effective methods exist to examine the ability of the tumor mass to migrate to surrounding tissues *in vitro*. We used spheres formed in 3D culture to investigate whether the migratory ability of tumors of PDAC cell lines, including epithelial and mesenchymal cell lines, varies.

**Methods:** Sphere formation and adhesion and spread on culture plates were examined by artificial intelligence-based analysis of time-lapse imaging using five epithelial and three mesenchymal PDAC cell lines. Fused and non-fused areas of the sphere surface during sphere formation on low-attachment plates, the adhesion area to normal culture plates, and the sphere area maintaining its original form during adhesion to plates were measured.

**Results:** Immunocytochemical staining confirmed that E-cadherin was highly expressed in epithelial PDAC spheres, as was vimentin in mesenchymal PDAC spheres, in 2D culture. When forming spheres using low-attachment plates, most epithelial PDAC cell lines initially showed decreased sphere area, and then the covering cells fused to form a smooth surface on the sphere. Mesenchymal PANC-1 and MIA PaCa-2 cells showed little reduction in sphere area and few areas of sphere surface fusion. When formed PDAC spheres were seeded onto normal culture plates, spheres of epithelial PK-8 cells—which have the highest E-cadherin expression, form numerous cysts, and have smooth sphere surfaces—did not adhere to normal plates even after 60 h, and epithelial PK45-P and T3M-4 spheres hardly adhered. Conversely, the area of adhesion and spread of mesenchymal PANC-1 and KP4 cell spheres on normal plates markedly increased from early on, forming large areas of attachment to plates.

**Conclusion:** Seeding spheres formed in 3D culture onto culture plates can clarify differences in tumor migration potential to surrounding areas. The masses formed by each PDAC cell line varied in migratory ability, with mesenchymal PDAC masses being more migratory than epithelial PDAC masses.

## 1 Introduction

Pancreatic cancer is a refractory cancer with an extremely low 5-year survival rate of approximately 12% (Siegel et al., 2023). In the 1990s, the survival rate for pancreatic cancer was around 3%–4% (Itakura et al., 1997; Parker et al., 1997; Kleeff et al., 1998) and has only slightly improved in the last quarter of a century, despite medical advances. In the United States, it is projected that by 2023, there will be 64,050 cases of pancreatic cancer and 50,550 related deaths (Siegel et al., 2023). By 2030, pancreatic cancer is expected to become the second leading cause of cancer-related deaths after lung cancer (Rahib et al., 2014), with an increasing number of cases and deaths in both men and women (Cronin et al., 2022). More than 90% of cases of pancreatic cancer are pathologically classified as pancreatic ductal adenocarcinomas (PDACs) (Jemal et al., 2008).

Gene expression in PDAC cells is diverse. Collisson et al. (2011) classified PDAC tissues into three subtypes: classical, quasi-mesenchymal, and exocrine-like subtypes, using transcriptional profile analysis. However, exocrine subtypes were not detected in PDAC cell lines (Collisson et al., 2011). The classical subtype is characterized by high expression of adhesion-associated and epithelial genes, whereas the quasi-mesenchymal subtype highly expresses mesenchyme-associated genes. The exocrine-like subtype showed relatively high expression of tumor cell-derived digestive enzyme genes. However, the subtype classifications were questioned because the exocrine-like subtype may have been caused by the contamination of normal acinar cells in the tumor tissues (Puleo et al., 2018). PDAC cell lines in 2-dimensional (2D) culture can be divided into the following: epithelial cell lines that express high levels of the epithelial marker E-cadherin and low levels of the mesenchymal marker vimentin and mesenchymal cell lines that show the opposite expression pattern (Shichi et al., 2019; Minami et al., 2021). In three-dimensional (3D) culture, which is supposed to mimic the *in vivo* environment, epithelial PDAC spheres had a circular, smooth surface and were covered with flat-lining cells. In contrast, mesenchymal PDAC cell spheres had an irregular spherical surface, and a few individual cancer cells were fused. Furthermore, the effects of anticancer drugs differ between epithelial and mesenchymal PDAC cell spheres (Minami et al., 2021); gemcitabine and nab-paclitaxel were effective for epithelial PDAC spheres and mesenchymal PDAC spheres, respectively.

In the human pancreas, PDAC cells form a tumor mass, which migrates to surrounding tissues, invades blood or lymph vessels, and metastasizes (Giovannetti et al., 2017; Murphy et al., 2022). Although the migratory and invasive capacities of individual PDAC cells have been studied using Boyden chamber or modified Boyden chamber methods (Minami et al., 2021), the adhesive and migratory capacities of the tumor mass, which comprises clumps of PDAC cells, have not been studied (Bouchalova and Bouchal, 2022). Therefore, this study aimed to investigate the adhesive and migratory capacities of spheres from epithelial and mesenchymal PDAC cells *in vitro*.

Herein, we confirmed that epithelial–mesenchymal features of PDAC cells observed in adhesion culture were maintained in the spheres and that the process of sphere formation differed between epithelial and mesenchymal PDAC cells. When the formed spheres were transferred to normal culture plates, mesenchymal PDACs tended to adhere earlier and more extensively than epithelial PDACs. This novel *in vitro* experimental method may provide insights into the ability of PDAC tumor masses to migrate to surrounding tissues in the human pancreas.

## 2 Materials and methods

### 2.1 Cell culture

PK-8, PK-45P, and T3M-4 human epithelial PDAC and KP4 mesenchymal PDAC cell lines were provided by the RIKEN BRC through the National Bio-Resource Project of the MEXT/AMED, Japan. Human epithelial PDAC cell lines PK-59 and PK-1 and mesenchymal PDAC cell lines PANC-1 and MIA PaCa-2 were obtained from the Cell Resource Center for Biomedical Research, Institute of Development, Aging and Cancer, Tohoku University (Sendai, Japan) (Sasaki et al., 2019a). Cells were grown in a growth medium (RPMI-1640 medium containing 10% fetal bovine serum) at 37°C in a humidified 5% CO_2_ atmosphere. Using the *Mycoplasma* PCR Detection Kit (iNtRON Biotechnology Inc., Jungwon-Gu, South Korea), we confirmed that none of the cells had *mycoplasma* contamination. Genomic DNA was extracted from PDAC cells using a DNeasy Blood and Tissue Kit (Qiagen, Hilden, Germany) according to the manufacturer’s instructions. Short tandem repeats were analyzed using the GenePrint 10 System (Promega, Madison, WI, United States of America), as described by the manufacturer. All PDAC cell lines were genotyped correctly and had no contamination.

### 2.2 Scanning electron microscopy

Scanning electron microscopy (SEM) analysis was performed with minor modifications of the methods reported previously (Ishiwata et al., 2018). PDAC spheres were fixed overnight with 2.5% glutaraldehyde in 0.1 M phosphate buffer (pH 7.4) at 4°C. The glutaraldehyde solution was then removed, and the cells were washed with phosphate-buffered saline. PDAC spheres were post-fixed with 1% OsO_4_ for 30 min. After complete dehydration using a graded ethanol series, the samples were suspended in 100% ethanol, air-dried, and coated with a platinum layer using an MSP-1S sputter coater (Shinku Device, Ibaraki, Japan). PDAC spheres were examined and photographed using a Phenom Pro desktop scanning electron microscope with reflected electrons (Thermo Fisher Scientific, Waltham, MA, United States of America) (Ishiwata et al., 2018; Sasaki et al., 2019b).

### 2.3 Immunocytochemical analysis

For sphere formation in 3D culture, PDAC cells in growth medium were plated at 3.0 × 10^3^ cells/well in 96-well low-attachment plates (Cat. No. 174925, Thermo Fisher Scientific). The spheres were aspirated after 7 days using micropipettes and used for immunocytochemistry. Cell blocks were prepared as previously reported (Shichi et al., 2019; Minami et al., 2021), with minor modifications. Briefly, PDAC spheres were fixed in formalin for 3 h at room temperature. Formalin was removed using a micropipette, and the spheres were dehydrated in graded ethanol and then embedded in paraffin. Subsequently, serial sections of the cell blocks (3-μm thickness) were stained using the Histofine Simple Stain Kit (Nichirei Biosciences Inc., Tokyo, Japan). Antigen retrieval was performed using the retrieved antigen solution. The following primary antibodies were used for immunocytochemical staining: mouse monoclonal anti-E-cadherin (M106; Takara Bio, Shiga, Japan) and mouse monoclonal anti-vimentin (422101; Nichirei). The sections were treated with 0.3% H_2_O_2_ in water at room temperature for 5 min to block endogenous peroxidase activity. Reactions to each antigen were visualized by the addition of 3,3-diaminobenzidine tetrahydrochloride and counterstaining with hematoxylin. Negative controls were generated by omitting primary antibodies. Images were captured using a Matra 2 multispectral microscope (PhenoImager Mantra2, AKOYA Biosciences, Marlborough, MA, United States of America) (Shichi et al., 2022b).

### 2.4 Time-lapse image acquisition

To form spheres, PDAC cells (3  ×  10^3^ cells/well) were plated in 96-well low-attachment plates (Thermo Fisher Scientific) with growth medium for 7 days. The PDAC spheres were then aspirated using micropipettes and transferred to 96-well normal culture plates (Cat. No. 167008, Thermo Fisher Scientific) with growth medium for 60 h. Time-lapse images of the process of sphere formation in the low-attachment plates and adhesion and spreading of the formed spheres to the normal plates were taken with a BZ-X710 fluorescence microscope (Keyence, Osaka, Japan) for 60 h at 15-min intervals for each cell. A 10x magnification objective lens was used to capture the sphere formation process, and a 4x objective lens was used to capture the adhesion and migration processes.

### 2.5 Measuring sphere area using artificial intelligence

**FIGURE 1:**
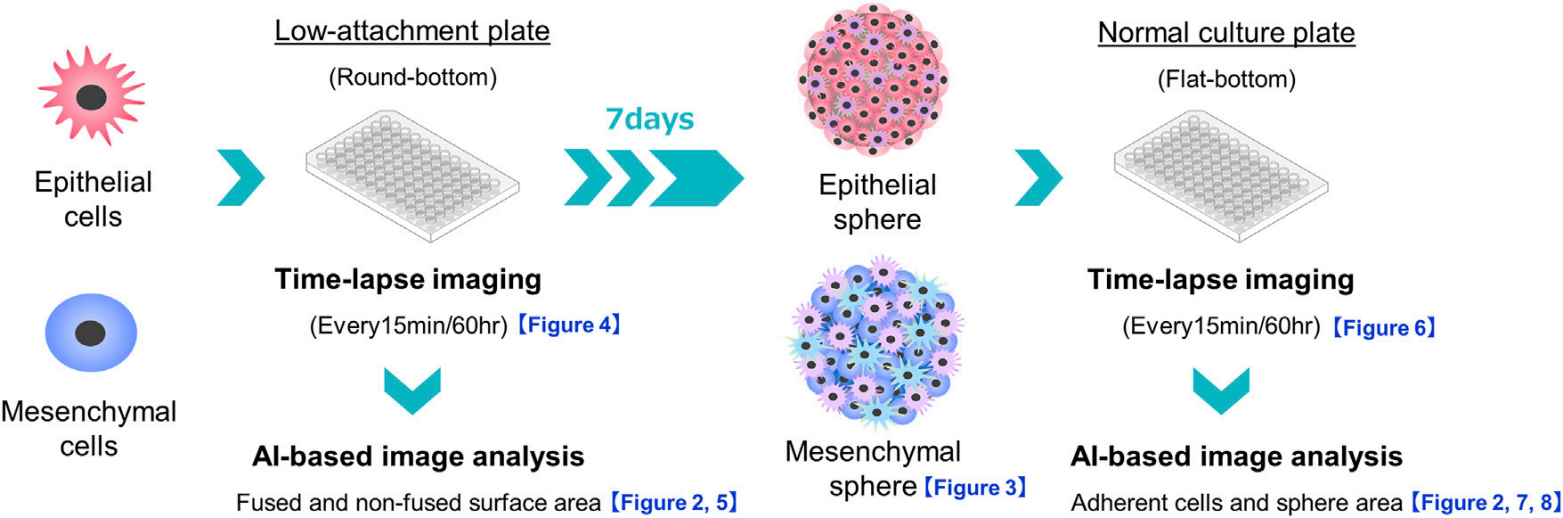
Schema of experiments on sphere formation and adhesion and spread of the formed spheres on normal culture plates. The sphere formation process was seeded at 3,000 cells/well of PDAC cells on a round-bottom low-adhesion plate, and time-lapse images were taken every 15 min for 60 h. The acquired images were divided into fused and non-fused areas using artificial intelligence (AI), and each area was measured. For sphere adhesion and migration, spheres were seeded in the same plate at 3,000 cells/well, and the formed spheres were collected after 1 week. The spheres were transferred to a flat-bottomed normal culture plate and time-lapse images were taken under the same conditions. The acquired images were divided by AI into the area of the original sphere and the area of cells that had adhered and migrated to the bottom of the plate, and each area was measured.

**FIGURE 2:**
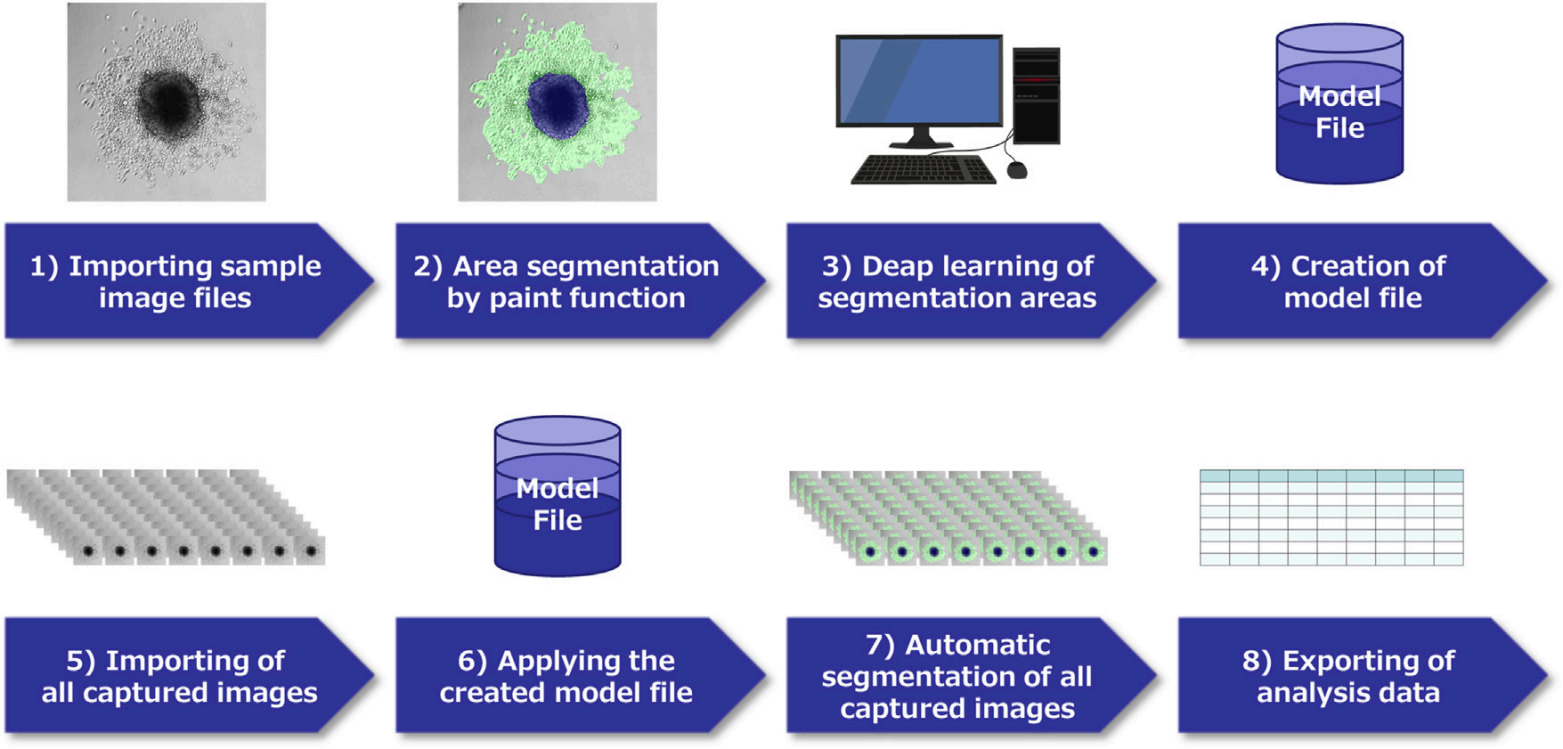
Schema of image analysis using a deep learning method. 1) In each pancreatic ductal adenocarcinoma (PDAC) cell captured by time-lapse imaging, select characteristic images and capture them. 2) Classify the region of each image by paint function. 3) Apply the AI deep learning method to the segmented images, and 4) Create a model file. 5) Import all acquired images of this experiment, 6) Analyze using the model file, 7) Perform automatic segmentation, 8) Extract the obtained data.

Using the obtained time-lapse images of spheres cultured in 96-well low-attachment plates (240 time-lapse images per PDAC sphere) and normal 96-well plates (240 time-lapse images per PDAC sphere), the areas of the spheres were evaluated using the Cell3iMager duos2 software (SCREEN Holdings Co., Ltd., Kyoto, Japan). The fused and non-fused areas on the surfaces of the spheres were separated and calculated using the software artificial intelligence (AI)-based deep learning methods (Saeki et al., 2021). The outline of this experiment is illustrated in Figure 1. Preliminary experiments were performed to identify appropriate time-lapse imaging conditions, and then all 10 time-lapse images of sphere formation and adhesion/migration were visually observed for each PDAC cell line. The formation process and the adhesion and migration processes were similar among the 10 spheres of each PDAC cell line. We then used the AI deep learning method to measure the specified area and plotted the average of the measurements for each of the three spheres (1,440 time-lapse images/1 cell line to form spheres and spread on culture plates). To ensure the accuracy of the measurements, the SCREEN deep learning tool V2.6 was used to enable continuous learning until the error index, LOSS, was 0.0429 and accuracy, which indicates the percentage of correct measurements per pixel, was 0.9820. A model file was then created (Figure 2). The areas of the PDAC cells that remained as spheres and those that adhered to the culture plates were measured in the same manner. Epithelial PK-8 cells were analyzed for the process of cyst formation by examining the relationship between sphere area alterations and time-lapse imaging.

## 3 Results

### 3.1 Immunocytochemical analyses of E-Cadherin and vimentin in PDAC spheres

**FIGURE 3:**
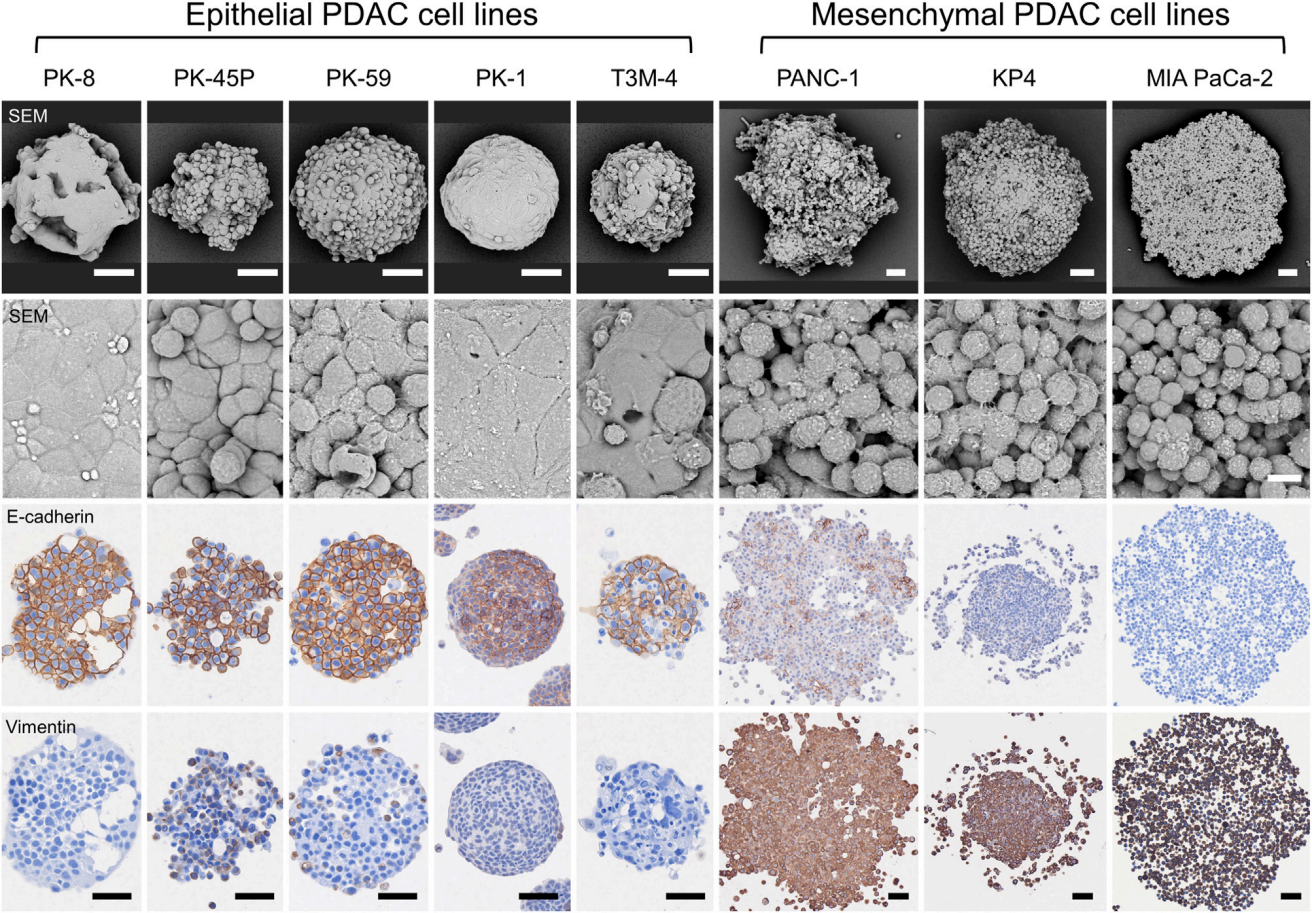
Scanning electron microscopy (SEM) and immunocytochemical analyses of human pancreatic ductal adenocarcinoma (PDAC) spheres. PDAC cell lines were cultured in 96 well low-attachment plates for 7 days, and the formed spheres were examined using SEM and immunocytochemical analyses for E-cadherin and vimentin. The spheres of each PDAC cell line are small and have a smooth surface in epithelial cell lines, whereas mesenchymal cell lines are large and cell-independent (first and second panels). E-cadherin protein is strongly localized to the sphere of epithelial PDAC cells, and vimentin protein is present in mesenchymal PDAC cells (third and fourth panels). Immunocytochemical staining for E-cadherin and vimentin was performed on serial tissue sections. Scale bars: second panels = 10 µm; all other panels = 50 μm.

SEM observation of the spheres in 3D culture showed that epithelial PDACs formed small spherical spheres with flattened coating cells on the sphere surface, whereas mesenchymal PDACs formed large spheres with small, similarly shaped cancer cells (Figure 3, first and second panels) (Shichi et al., 2019; Minami et al., 2021; Shichi et al., 2022a). In 3D culture, E-cadherin was strongly localized to the cell membrane of most cells forming epithelial spheres and weakly localized to the cell membrane of a few cells in mesenchymal PANC-1 spheres but not in those of KP4 and MIA PaCa-2 cell spheres (Figure 3, third panel). In contrast, vimentin was strongly expressed in the cytoplasm of most cells forming mesenchymal spheres (Figure 3, fourth panel). There was no vimentin localization in the spheres of epithelial PK-8, PK-1, and T3M-4 cells; however, weak localization was observed in a few epithelial PK-45P and PK-59 cells that formed spheres. These expression patterns were similar to those observed in 2D cultures of epithelial and mesenchymal PDAC cells (Shichi et al., 2019; Minami et al., 2021).

### 3.2 Time-lapse images of PDAC sphere formation

**FIGURE 4:**
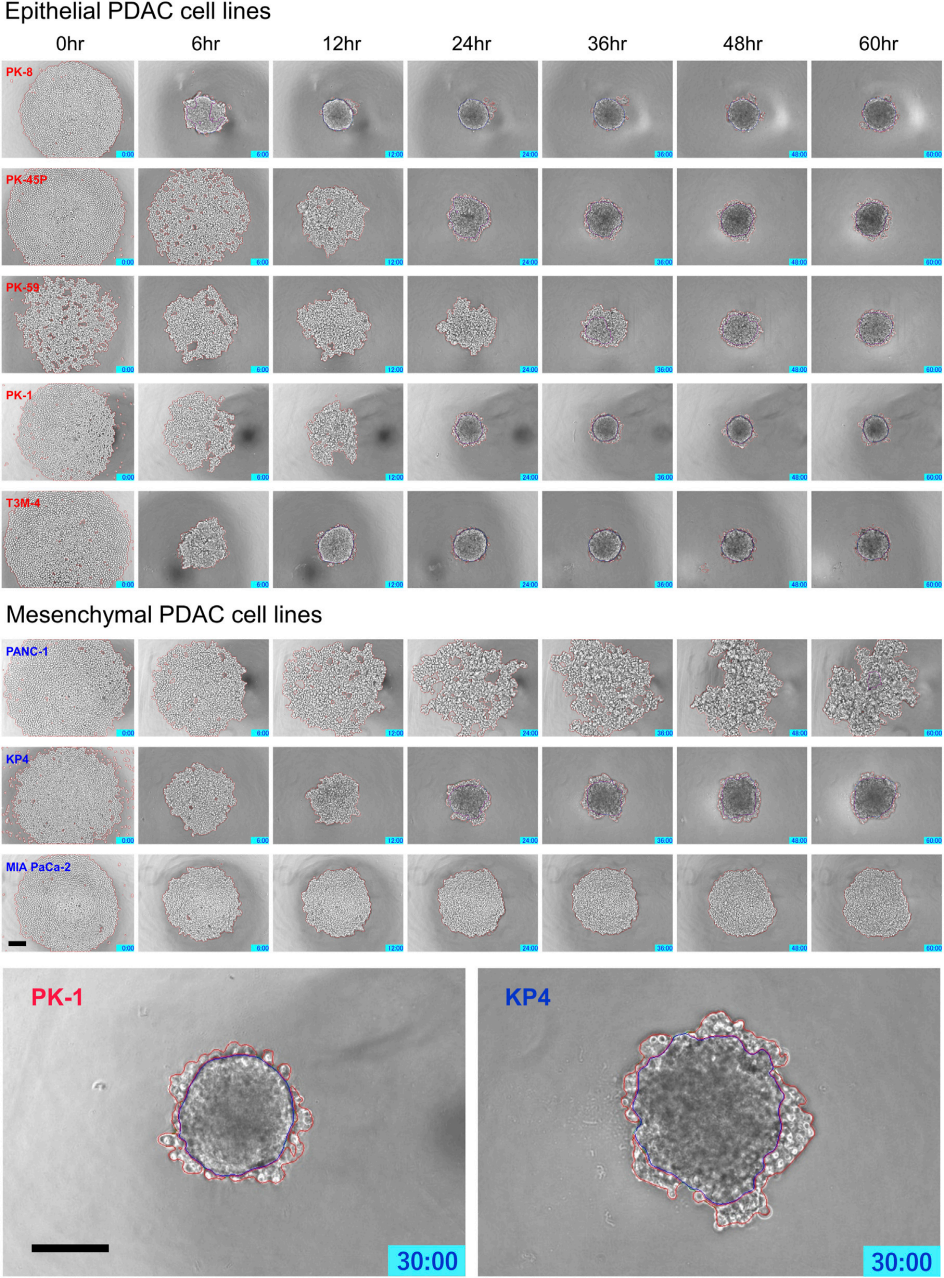
Characteristic time-lapse images of sphere formation in human pancreatic ductal adenocarcinoma (PDAC) cell lines. Five epithelial and three mesenchymal PDAC cell lines were cultured for 60 h on 96 well low-attachment plates (round-bottom). Two hundred and forty time-lapse images were obtained per sphere, and three spheres were obtained for each cell line. Epithelial PDAC cell lines formed small round spheres early (upper 5 panels), whereas mesenchymal PDAC cell lines formed large spheres with irregularly shaped margins (middle 3 panels). Sphere areas circled in blue are fused areas, and areas circled in red are non-fused areas (lowest panels). Scale bar = 200 μm.

**FIGURE 5:**
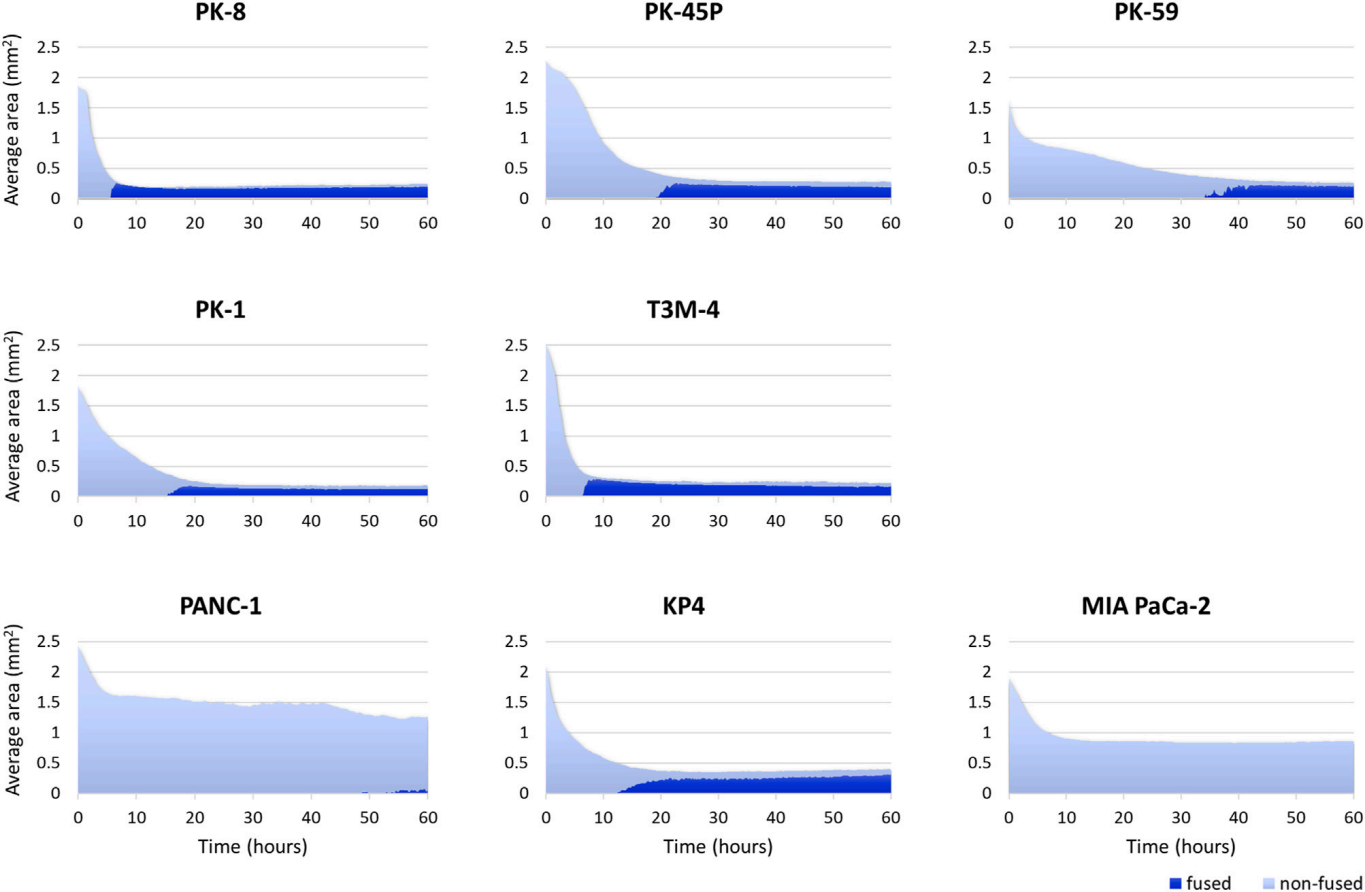
Artificial intelligence (AI)-based analysis of fused and non-fused areas during human pancreatic ductal adenocarcinoma (PDAC) cell line sphere formation. Two hundred and forty time-lapse images per sphere were obtained, and three spheres for 1 cell line (720 images in total) were analyzed using an AI-based deep learning method. The figure shows the average values of the three spheres. All epithelial PDAC cell lines and mesenchymal KP4 cells showed an immediate decrease in sphere area, followed by an increase in fusion area. Dark blue: fused area, light blue: non-fused area.

Time-lapse images showed that epithelial PDAC cell lines, including PK-8, PK-45P, PK-59, PK-1, and T3M-4, formed small round spheres early (Figure 4, upper five panels: Supplementary Movies S1, S2). In contrast, mesenchymal PDACs, PANC-1, KP4, and MIA PaCa-2 cells formed large spheres with irregularly shaped margins (Figure 4, lower 3 panels: Supplementary Movies S3, S4). The areas of cell fusion (circled in blue) and non-fusion on the surfaces of the spheres (circled in red) were determined using an AI-based deep learning method (Figure 4). All epithelial PDAC cell lines and mesenchymal KP4 cells exhibited increased fusion areas over time (Figure 5, dark blue). For epithelial PK-8 and T3M-4 cells, which were the fastest surface cells to fuse, cell fusion began after 5 h, and for epithelial PK-59 cells, which were the slowest cells to fuse, cell fusion began after 35 h. In contrast, the spheres of mesenchymal PANC-1 cells showed little surface cell fusion, whereas MIA PaCa-2 cells showed no areas of cell fusion.

### 3.3 Time-lapse analyses of adhesion and spreading of human PDAC spheres in culture plates

**FIGURE 6:**
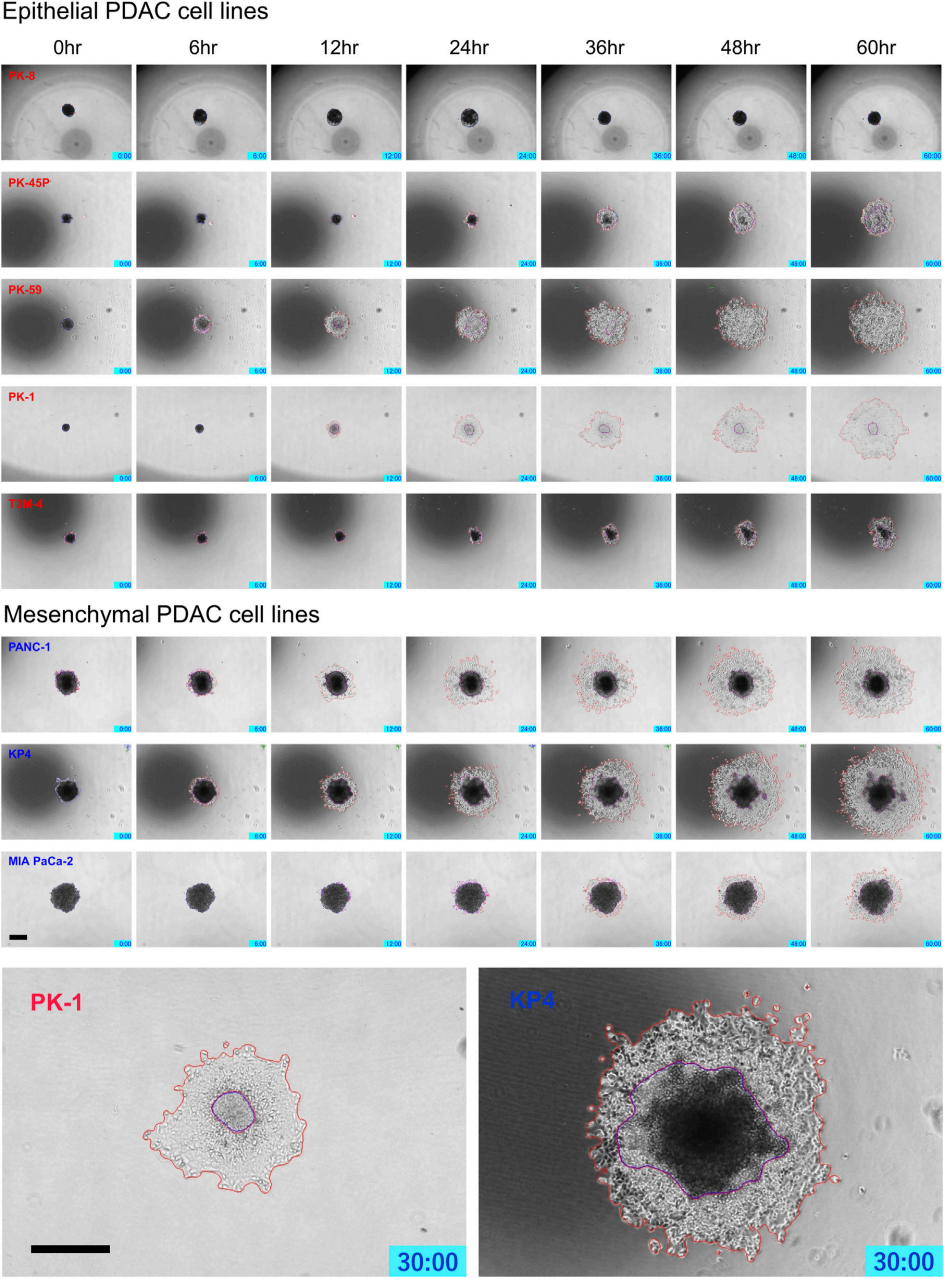
Characteristic time-lapse images of the adhesion and spread of human pancreatic ductal adenocarcinoma (PDAC) spheres on normal culture plates. Spheres of PDAC cells formed by 96-well low-attachment plates for 7 days were transferred to 96-well normal plates. For all PDAC cell lines, except for PK-8, the spheres adhered to the bottom of the normal culture plates, and the epithelial cell lines, except the PK-8 cell line, migrated in sheets with cells adhering to each other. In mesenchymal PDAC cell lines, each cell migrated individually and radially. The blue area represents the area of PDAC spheres, and the red area represents the area of the radially spread cells adhered to the plate. Scale bar = 500 μm.

**FIGURE 7:**
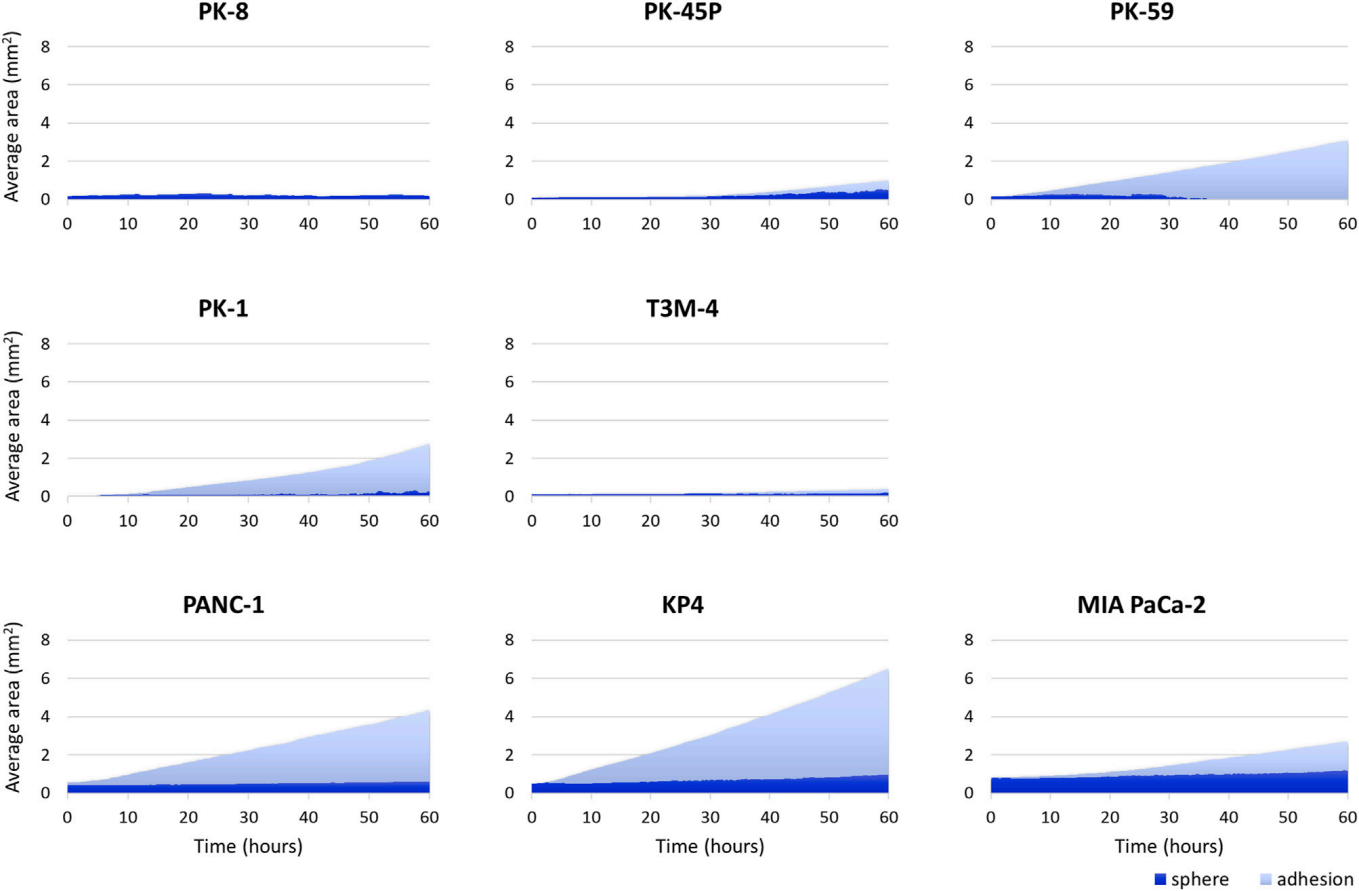
Artificial intelligence (AI)-based analysis of the areas of the human pancreatic ductal adenocarcinoma (PDAC) spheres and cells attached and spread on the plates. The graph shows the area of spheres in each PDAC cell and the area where the cells adhered to and spread on the normal plates. Two hundred and forty time-lapse images per sphere were analyzed using an AI-based deep learning method; three spheres were examined for each cell line, and the average values are shown. Epithelial PK-8 cells did not adhere to the culture plates during the observed 60 h. PK-45P and T3M-4 cell spheres of epithelial PDAC cells adhered to the bottom of the plate, but barely spread to the surrounding area. Mesenchymal KP4 cells and PANC-1 cells were abundantly adherent and spread on the culture plates. Dark blue: the sphere area, and light blue: the adhesion area to the bottom of the plates.

PDAC spheres formed on 96-well low-attachment plates were transferred to normal 96-well plates, and adhesion and spreading to the bottom of the plates were analyzed using time-lapse images. For all PDAC cell lines, except for PK-8, the spheres adhered to the bottom of the normal culture plates, and the cancer cells spread radially over the plate (Figure 6, Supplementary Movies S5–S8). The epithelial cell lines, except for the PK-8 cell line, migrated in sheets with cells adhering to each other, whereas in mesenchymal cell lines, each cell migrated individually and radially in a plate-like fashion (Figure 6, Supplementary Movies S5–S8). The AI-based deep learning method was used to measure the area that was attached to the plate (Figure 6, areas circled in red) and the area that remained as spheres (areas circled in blue) using 240 time-lapse images per sphere. Epithelial PK-8 cells did not adhere to the culture plates during the observed 60 h (Figure 7). Although epithelial PK-45P and T3M-4 cell spheres of epithelial PDAC cells adhered to the bottom of the plate, the cancer cells barely spread to the surrounding area. Mesenchymal KP4 cells were the most abundant, and PANC-1 cells were the second most abundant cells spread across the culture plate. In mesenchymal PDAC cells, the area of the spheres remained almost constant, but the area attached to the plate increased.

### 3.4 Time-lapse analysis of repeated cyst rupture of PK-8 cell spheres

**FIGURE 8:**
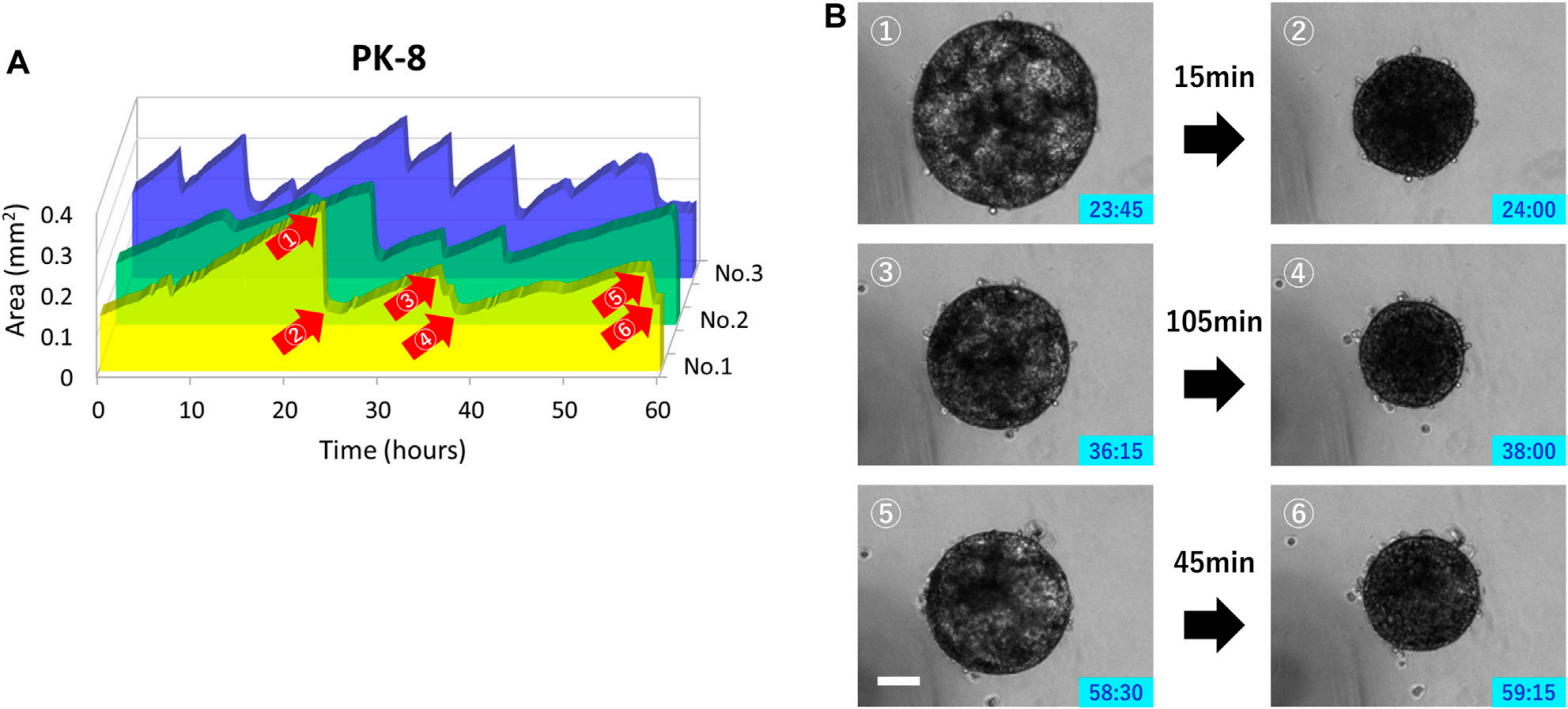
Rupture and re-expansion of floating epithelial PK-8 cell spheres in normal plates. Sphere areas of PK-8 cells floating in the normal plates were measured using an artificial intelligence-based deep learning method. The three wavy lines indicate that the area of the spheres (n = 3) was rapidly decreasing and repeating a gradual increase **(A)**. Clear cystic areas are observed in the expanded PK-8 cell spheres (1, 3, and 5), but not in the spheres that have exhibited decreased area (2, 4, and 6) **(B)**. The numbers (1) through (6) in Figure A correspond to the pictures (1) through (6) in Figure **(B)**. Scale bars = 20 μm.

Only PK-8 cells, which had the highest E-cadherin expression among epithelial PDAC cells, did not adhere to normal culture plates and floated in the culture medium. Thus, we examined the relationship between time-lapse images and sphere area in detail. In three PK-8 spheres, a wavy line representing a sudden and large change in area was observed after adjusting the area scale of the graph (Figure 8A). When the spheres became smaller, the transparent areas within the spheres, which were indicative of cysts (Shichi et al., 2022b), disappeared at least three times during the 60-h period (Figure 8B: Supplementary Movie S5). These analyses indicate that the rapid decrease in the sphere area of the frequent PK-8 cells was due to the rupture of dilated cysts.

## 4 Discussion

We have previously reported that in 2D cultures, epithelial PDAC cell lines, including PK-8, PK-45P, PK-59, PK-1, and T3M-4, were E-cadherin-positive and vimentin-negative or weakly positive. In contrast, mesenchymal PDAC cells, including PANC-1, KP4, and MIA PaCa-2, showed the opposite expression pattern in immunocytochemical analysis (Minami et al., 2021)*.* In the present study, immunostaining of spheres in 3D culture performed showed a similar tendency to that in 2D culture, with E-cadherin localizing more strongly to the cell membrane of epithelial cancer cells, and vimentin localizing more strongly to the cytoplasm of mesenchymal cancer cells. However, unlike 2D cultures, a small number of 3D cultured mesenchymal PANC-1 cells expressed E-cadherin at the cell membrane. The slight expression of vimentin detected in epithelial PK-45P cells in 2D culture became more evident in 3D culture and was also detected in PK-59 cells. These findings indicate that epithelial–mesenchymal features are retained in PDAC cells even in 3D culture and that some PDAC cells may have both properties, although one is dominant.

We have also previously reported that epithelial PDAC cell lines form small round spheres with flat surfaces (Shichi et al., 2019; Minami et al., 2021; Shichi et al., 2022a). In contrast, the mesenchymal PDAC cell lines formed large spheres; the cells on the surface of the spheres did not adhere to each other and maintained their original shape. Pathologically, a smooth surface of the mass suggests a benign feature, whereas an irregular surface is considered to indicate malignant properties and is responsible for the tumor’s ability to migrate and metastasize to surrounding tissues. The morphology of 3D cultured cell lines is variable and is related to their prognosis (Han et al., 2010). In this study, time-lapse imaging of sphere formation was performed to determine the regions of cell fusion using a machine learning function. Most epithelial PDAC cell lines showed area reduction and cell-to-cell fusion within 24 h. In contrast, mesenchymal PDAC cells, PANC-1 and MIA PaCa-2, showed only a slight decrease in area and few fusion regions in the early phase. Notably, epithelial PK-59 cells, which express vimentin in 3D culture, showed a slow decrease in area and slow formation of fusion regions. KP4 cells, which form the smallest spheres in the mesenchyme, showed a rapid decrease in area and formation of fusion regions. These sphere formation processes indicate diversity among epithelial and mesenchymal PDAC cells.

In our previously reported modified Boyden chamber assay with a Matrigel-coated chamber using 2D cultured PDAC cells, mesenchymal PANC-1 cells showed the highest migration ability, and mesenchymal KP4 and PANC-1 cells showed extremely high invasion abilities (Minami et al., 2021). Although these assays can examine the migratory and invasive capacities of individual cells, in the human body, cancer cells form a mass at the primary site and then migrate to the surrounding area. Therefore, we examined how spheres, a mass of cancer cells, spread on a plate when attached to a normal culture plate and clarified the differences in adhesion and spreading among PDAC cells. Notably, PK-8 cells, which are epithelial PDAC cells with the highest E-cadherin mRNA expression and form many cysts in the sphere (Shichi et al., 2022b), did not adhere to the normal plate for 60 h. In addition, the epithelial PK-45P and T3M-4 spheres showed little adhesion and spread on the plate, whereas the mesenchymal PANC-1 and KP4 spheres rapidly adhered and increased their adherent area without mutual fusion. PANC-1 and KP4 spheres have higher adhesion and spreading abilities than MIA PaCa-2 spheres, similar to an assay of individual cells (Minami et al., 2021). Spheres of epithelial PDAC cells tended to be less likely to adhere to the plate and spread, whereas mesenchymal PDAC spheres tended to adhere and spread more quickly. In epithelial cell lines, except for the PK-8 cell line, the migrating cells adhered to each other and spread out in sheets, whereas, in the mesenchymal cell line, each cell spread out individually and radially. Differences in the migration ability and individual cell behavior among cell lines have long been noted (Deer et al., 2010), suggesting that cells from spheres have similar abilities in the adhesion–migration process. Future investigations should analyze how PDAC cell spheres adhere and migrate in the presence of scaffolds, such as extracellular matrix, to understand their ability to invade cancer. In addition to cultured cell lines, it is also necessary to examine migration and invasion using primary cultured cells and organoids.

Transmission electron microscopy and optical coherence tomography observations of 3D-cultured PK-8 cells revealed the formation of numerous cysts of various sizes inside the spheres (Shichi et al., 2022b). In the present study, 3D-cultured PK-8 cell spheres expressed mucins MUC1 and MUC5AC, which are characteristics of PDAC cells, and amylase. PK-8 cells forming multicystic spheres did not adhere to the plate, and in the time-lapse images, the transparent cystic portion of the spheres repeatedly disappeared and swelled. Taken together, the rapid decrease in the area of PK-8 spheres suggests that the cysts ruptured and re-expanded repeatedly. Pancreatic cysts are a risk factor for the development of pancreatic cancer. The risk of malignant transformation of incidental pancreatic cystic lesions is low (0.01%) but increases to 0.21% for cysts >2 cm (Lira-Treviño et al., 2022). Therefore, an accurate diagnosis is essential for the management of pancreatic cysts. In addition, PK-8 cells formed cysts in 3D culture using low-attachment plates without any matrices, and the size of the cysts did not change because of repeated cyst rupture and expansion. Instead of a benign lesion turning into a malignant PDAC, some pancreatic cysts may be formed by PDAC cells themselves. Therefore, a qualitative diagnosis of pancreatic cysts is important although they are small and may not change in size during the follow-up period.

The tumor cell evaluation technique in this study is a technology that may bridge the gap between two-dimensional migration ability and *in vivo* evaluation. However, to render this technique widely applicable in studies such as the response to anticancer drugs, it is necessary to enable high-throughput analysis by introducing automation. We have previously attempted to induce mesenchymal to epithelial lineage by introducing the *epithelial splicing regulatory 1* (*ESRP1*) gene into a PDAC cell line (Ueda et al., 2014). PDAC cells induced into the epithelial lineage showed reduced migration ability in 2D culture and suppressed tumor metastasis in animal experiments. Moreover, TGF-β1 induces epithelia-mesenchymal transition in pancreatic cancer culture lines (Shichi et al., 2019), and our method can also be used to evaluate alterations in the adhesion and migration abilities due to the shift from epithelial to mesenchymal traits.

## 5 Conclusion

The epithelial–mesenchymal features in PDAC cells were retained in 3D cultures. Four of the five epithelial PDAC spheres showed a rapid decrease in area, followed by an increase in the area of cellular fusion on the sphere surface, whereas two of the three mesenchymal PDAC spheres showed little decrease in area and few areas of surface adhesion. Compared with epithelial PDAC spheres, mesenchymal PDAC spheres showed marked adhesion and spread on the plate. Epithelial PK-8 cell spheres with the highest E-cadherin expression did not adhere to the normal culture plate with repeated cyst rupture and dilatation. These findings indicate that this novel experimental method may provide valuable insights into the ability of PDAC tumor masses to migrate to surrounding tissues in the human pancreas and may assist the development of therapies to suppress migration.

## Data Availability

The datasets presented in this study can be found in online repositories. The names of the repository/repositories and accession number(s) can be found in the article/Supplementary Material.

## Abbreviations

2D, 2-dimensional; 3D, 3-dimensional; AI, artificial intelligence; PDAC, pancreatic ductal adenocarcinoma; SEM, scanning electron microscopy.

## References

[B1] Bouchalova P. Bouchal P. (2022). Current methods for studying metastatic potential of tumor cells. Cancer Cell Int. 22, 394. 10.1186/s12935-022-02801-w 36494720 PMC9733110

[B2] Collisson E. A. Sadanandam A. Olson P. Gibb W. J. Truitt M. Gu S. (2011). Subtypes of pancreatic ductal adenocarcinoma and their differing responses to therapy. Nat. Med. 17, 500–503. 10.1038/nm.2344 21460848 PMC3755490

[B3] Cronin K. A. Scott S. Firth A. U. Sung H. Henley S. J. Sherman R. L. (2022). Annual report to the nation on the status of cancer, part 1: national cancer statistics. Cancer 128, 4251–4284. 10.1002/cncr.34479 36301149 PMC10092838

[B4] Deer E. L. González-Hernández J. Coursen J. D. Shea J. E. Ngatia J. Scaife C. L. (2010). Phenotype and genotype of pancreatic cancer cell lines. Pancreas 39, 425–435. 10.1097/MPA.0b013e3181c15963 20418756 PMC2860631

[B5] Giovannetti E. van der Borden C. L. Frampton A. E. Ali A. Firuzi O. Peters G. J. (2017). Never let it go: stopping key mechanisms underlying metastasis to fight pancreatic cancer. Semin. Cancer Biol. 44, 43–59. 10.1016/j.semcancer.2017.04.006 28438662

[B6] Han J. Chang H. Giricz O. Lee G. Y. Baehner F. L. Gray J. W. (2010). Molecular predictors of 3D morphogenesis by breast cancer cell lines in 3D culture. PLOS Comput. Biol. 6, e1000684. 10.1371/journal.pcbi.1000684 20195492 PMC2829039

[B7] Ishiwata T. Hasegawa F. Michishita M. Sasaki N. Ishikawa N. Takubo K. (2018). Electron microscopic analysis of different cell types in human pancreatic cancer spheres. Oncol. Lett. 15, 2485–2490. 10.3892/ol.2017.7554 29434962 PMC5777357

[B8] Itakura J. Ishiwata T. Friess H. Fujii H. Matsumoto Y. Büchler M. W. (1997). Enhanced expression of vascular endothelial growth factor in human pancreatic cancer correlates with local disease progression. Clin. Cancer Res. 3, 1309–1316. 9815813

[B9] Jemal A. Siegel R. Ward E. Hao Y. Xu J. Murray T. (2008). Cancer statistics, 2008. CA Cancer J. Clin. 58, 71–96. 10.3322/CA.2007.0010 18287387

[B10] Kleeff J. Ishiwata T. Kumbasar A. Friess H. Büchler M. W. Lander A. D. (1998). The cell-surface heparan sulfate proteoglycan glypican-1 regulates growth factor action in pancreatic carcinoma cells and is overexpressed in human pancreatic cancer. J. Clin. Invest. 102, 1662–1673. 10.1172/JCI4105 9802880 PMC509114

[B11] Lira-Treviño A. Carranza Mendoza I. G. Borbolla Arizti J. P. Soriano-Ríos A. Uscanga-Domínguez L. Peláez-Luna M. (2022). Pancreatic cystic lesions. Differential diagnosis and treatment strategy. Rev. Gastroenterol. Mex. Engl. Ed. 87, 188–197. 10.1016/j.rgmxen.2022.05.002 35610168

[B12] Minami F. Sasaki N. Shichi Y. Gomi F. Michishita M. Ohkusu-Tsukada K. (2021). Morphofunctional analysis of human pancreatic cancer cell lines in 2- and 3-dimensional cultures. Sci. Rep. 11, 6775. 10.1038/s41598-021-86028-1 33762591 PMC7990961

[B13] Murphy K. J. Zhu J. Trpceski M. Pereira B. A. Timpson P. Herrmann D. (2022). Focal adhesion kinase priming in pancreatic cancer, altering biomechanics to improve chemotherapy. Biochem. Soc. Trans. 50, 1129–1141. 10.1042/BST20220162 35929603 PMC9444069

[B14] Parker S. L. Tong T. Bolden S. Wingo P. A. (1997). Cancer statistics, 1997. CA Cancer J. Clin. 47, 5–27. 10.3322/canjclin.47.1.5 8996076

[B15] Puleo F. Nicolle R. Blum Y. Cros J. Marisa L. Demetter P. (2018). Stratification of pancreatic ductal adenocarcinomas based on tumor and microenvironment features. Gastroenterology 155, 1999–2013. 10.1053/j.gastro.2018.08.033 30165049

[B16] Rahib L. Smith B. D. Aizenberg R. Rosenzweig A. B. Fleshman J. M. Matrisian L. M. (2014). Projecting cancer incidence and deaths to 2030: the unexpected burden of thyroid, liver, and pancreas cancers in the United States. Cancer Res. 74, 2913–2921. 10.1158/0008-5472.CAN-14-0155 24840647

[B17] Saeki K. Chang G. Kanaya N. Wu X. Wang J. Bernal L. (2021). Mammary cell gene expression atlas links epithelial cell remodeling events to breast carcinogenesis. Commun. Biol. 4, 660. 10.1038/s42003-021-02201-2 34079055 PMC8172904

[B18] Sasaki N. Hirabayashi K. Michishita M. Takahashi K. Hasegawa F. Gomi F. (2019a). Ganglioside GM2, highly expressed in the MIA PaCa-2 pancreatic ductal adenocarcinoma cell line, is correlated with growth, invasion, and advanced stage. Sci. Rep. 9, 19369. 10.1038/s41598-019-55867-4 31852956 PMC6920443

[B19] Sasaki N. Toyoda M. Hasegawa F. Fujiwara M. Gomi F. Ishiwata T. (2019b). Fetal bovine serum enlarges the size of human pancreatic cancer spheres accompanied by an increase in the expression of cancer stem cell markers. Biochem. Biophys. Res. Commun. 514, 112–117. 10.1016/j.bbrc.2019.04.117 31027735

[B20] Shichi Y. Gomi F. Sasaki N. Nonaka K. Arai T. Ishiwata T. (2022a). Epithelial and mesenchymal features of pancreatic ductal adenocarcinoma cell lines in two- and three-dimensional cultures. J. Pers. Med. 12, 746. 10.3390/jpm12050746 35629168 PMC9146102

[B21] Shichi Y. Gomi F. Ueda Y. Nonaka K. Hasegawa F. Hasegawa Y. (2022b). Multiple cystic sphere formation from PK-8 cells in three-dimensional culture. Biochem. Biophys. Rep. 32, 101339. 10.1016/j.bbrep.2022.101339 36105614 PMC9464880

[B22] Shichi Y. Sasaki N. Michishita M. Hasegawa F. Matsuda Y. Arai T. (2019). Enhanced morphological and functional differences of pancreatic cancer with epithelial or mesenchymal characteristics in 3D culture. Sci. Rep. 9, 10871. 10.1038/s41598-019-47416-w 31350453 PMC6659675

[B23] Siegel R. L. Miller K. D. Wagle N. S. Jemal A. (2023). Cancer statistics, 2023. CA Cancer J. Clin. 73, 17–48. 10.3322/caac.21763 36633525

[B24] Ueda J. Matsuda Y. Yamahatsu K. Uchida E. Naito Z. Korc M. (2014). Epithelial splicing regulatory protein 1 is a favorable prognostic factor in pancreatic cancer that attenuates pancreatic metastases. Oncogene 33, 4485–4495. 10.1038/onc.2013.392 24077287 PMC4041859

